# Differential effect of CLK SR Kinases on HIV-1 gene expression: potential novel targets for therapy

**DOI:** 10.1186/1742-4690-8-47

**Published:** 2011-06-17

**Authors:** Raymond Wong, Ahalya Balachandran, Annie YQ Mao, Wendy Dobson, Scott Gray-Owen, Alan Cochrane

**Affiliations:** 1 Dept. of Laboratory Medicine & Pathobiology, University of Toronto. Ontario, Canada; 2 Dept. of Molecular Genetics, University of Toronto, Toronto, Ontario, Canada

## Abstract

**Background:**

RNA processing plays a critical role in the replication of HIV-1, regulated in part through the action of host SR proteins. To explore the impact of modulating SR protein activity on virus replication, the effect of increasing or inhibiting the activity of the Cdc2-like kinase (CLK) family of SR protein kinases on HIV-1 expression and RNA processing was examined.

**Results:**

Despite their high homology, increasing individual CLK expression had distinct effects on HIV-1, CLK1 enhancing Gag production while CLK2 inhibited the virus. Parallel studies on the anti-HIV-1 activity of CLK inhibitors revealed a similar discrepant effect on HIV-1 expression. TG003, an inhibitor of CLK1, 2 and 4, had no effect on viral Gag synthesis while chlorhexidine, a CLK2, 3 and 4 inhibitor, blocked virus production. Chlorhexidine treatment altered viral RNA processing, decreasing levels of unspliced and single spliced viral RNAs, and reduced Rev accumulation. Subsequent experiments in the context of HIV-1 replication in PBMCs confirmed the capacity of chlorhexidine to suppress virus replication.

**Conclusions:**

Together, these findings establish that HIV-1 RNA processing can be targeted to suppress virus replication as demonstrated by manipulating individual CLK function and identified chlorhexidine as a lead compound in the development of novel anti-viral therapies.

## Background

The dependence of HIV-1 replication on the appropriate balance of its RNA processing suggests that this step in the virus lifecycle might be an attractive target for therapeutic intervention [1-3]. From a single 9 kb transcript, over 40 mRNAs are generated by a process of suboptimal splicing that generates three classes of HIV-1 mRNAs: unspliced (US) RNA used to produce Gag and Gagpol proteins; singly spliced (SS) mRNAs encoding Vif, Vpr, Vpu or Env; and multiply spliced (MS) mRNAs used to synthesize Rev, Tat or Nef. Both Tat and Rev play central roles in the replication of HIV-1. Tat increases abundance of all viral RNAs by increasing elongation efficiency of RNA polymerase II on proviral DNA [4,5] while Rev promotes the transport of unspliced and singly spliced viral RNAs to the cytoplasm [6,7]. Consequently, factors which alter the extent of HIV-1 RNA splicing can have dramatic effects on the extent of viral replication; undersplicing resulting in the loss of Tat and Rev while oversplicing reduces the abundance of incompletely spliced RNAs so that there is insufficient Gag and Env protein for new virion assembly. Understanding how to manipulate conditions within the cell to alter the extent of HIV-1 RNA splicing could provide insights into new strategies to control this infection.

Studies to date have identified a number of cis- and trans-acting factors involved in regulating HIV-1 RNA splicing [1,2]. Examination of the four splice donors and eight splice acceptors, used in generating the complete spectrum of viral mRNAs, demonstrated that much of the regulation is due to the suboptimal nature of the sequences that comprise the 3' splice sites (3'ss). Mutations that optimize the splice sites result in dramatic shifts in usage, increasing the extent of viral RNA splicing and reducing HIV-1 replication [8,9]. Use of specific 3'ss is also regulated by the presence of exon splicing silencers (ESSs) and exon splicing enhancers (ESEs) that act in an antagonistic fashion to suppress or promote, respectively, the use of particular splice sites. The majority of HIV-1 ESSs function by binding of hnRNP A1, which promotes addition of further hnRNP A1 molecules to adjacent sequences and thereby sterically blocks interaction of U2 snRNP and U2AF with the branchpoint and polypyrimidine tract [10-14]. The ESEs counter the ESSs by the binding of specific members of the SR protein family. SR proteins consist of one or two N-terminal RNA binding motifs and a C-terminus rich in arginine-serine dipeptides which collaborate to promote the use of adjacent splice sites by stabilizing interaction of splicing factors (such as U2AF, U1 snRNP) with the splice site signals [15]. In some instances, binding to an ESE also occludes interaction of factors with the adjacent/overlapping ESS [13,16]. The significance of these factors in regulating HIV-1 RNA processing has been illustrated by examining the effect of mutating the cis elements in viral RNA or altering SR protein expression levels in cells. Mutations which inactivate the ESS near the *vpr *reading frame (ESSV) resulted in both a significant increase in use of the adjacent 3' splice site (splice acceptor 2, SA2) but also a marked decrease in unspliced viral RNA abundance leading to a loss of virus replication [17]. Similarly, mutations in Env have been identified that activate a cryptic splice through recruitment of the SR protein SRSF2 (SC35) and hnRNP H [18]. In addition, overexpression of the SR protein SRSF1(SF2/ASF) has been shown to increase use of the 3'ss for Vpr (SA2) while increased levels of SRSF2/SRFS7 (9G8) induce use of the 3'ss for Tat (SA3) [19-21].

The sensitivity of HIV-1 RNA processing to changes in abundance or activity of SR proteins has suggested that these factors could be targeted to achieve changes in the nature and/or extent of viral RNA splicing so as to inhibit HIV-1 replication. Support for this hypothesis can be found in the observation that HIV-1 infection is associated with changes in SR protein phosphorylation/abundance that could be reversed upon overexpression of SR protein kinase 2 (SRPK2) [22-24]. Several proteins have been shown to phosphorylate SR proteins, with members of the SR protein kinase (SRPK1 and SRPK2) and Cdc2 like kinase (CLK1, CLK2, CLK3 and CLK4) families being the most intensively studied [25]. Comparison of SRPK and CLK kinases have revealed that, while both can phosphorylate SR proteins, they differ in the extent of phosphorylation and in the protein sequences modified [26-29]. This fact, coupled with differences in subcellular localization (SRPKs are cytoplasmic while CLKs are nuclear), suggests that they play distinct roles in regulating SR protein activity [30-33].To explore the role of SR kinases other than SRPK2 in regulating HIV-1 gene expression, we examined the impact of overexpressing members of the CLK family on viral RNA abundance and protein synthesis. The four members of the CLK family (CLK1/Sty, CLK2, CLK3 and CLK4) have overlapping specificity for the phosphorylation of specific SR proteins [25]. Despite a significant degree of homology between the various CLK members, we observed that they had disparate effects on HIV-1 expression; CLK1 promoting expression of HIV-1 Gag while CLK2 dramatically suppressed synthesis of viral structural proteins. Effects at the protein level were mirrored in alterations in viral RNA abundance, suggesting that CLK1 and CLK2 act to modulate HIV-1 RNA processing in distinct ways. Based on these observations, we also explored the effects of recently described CLK inhibitors (TG003, chlorhexidine) on HIV-1 replication [34,35]. Similar to the results with the individual CLKs, we observed that the two inhibitors had markedly different effects on viral gene expression: TG003 treatment had no effect while chlorhexidine significantly suppressed HIV-1 Gag synthesis. In context of HIV-1 growth in PBMCs, chlorhexidine also suppressed virus replication. Given that chlorhexidine is currently used in humans at doses ~1000 fold greater than used in our assays, our findings suggest that this compound could be used at mucosal surfaces to prevent virus transmission.

## Results

### CLK1 Increases While CLK2 Decreases HIV-1 Gene Expression

**Figure 1:**
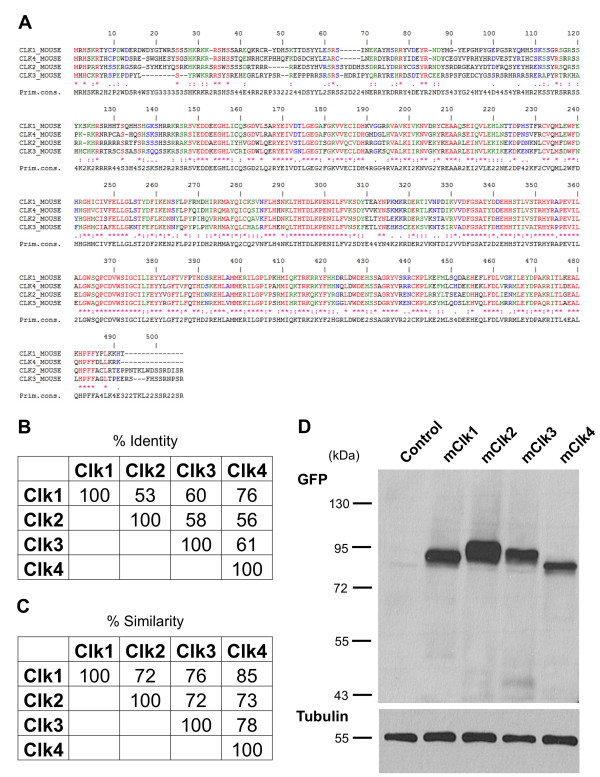
**Analysis of CLK Homology and Expression**. Sequence alignment of CLK 1, 2, 3 and 4 indicating the degree of homology among these factors. Also shown are the % sequence identity (B) and sequence similarity (C) among the four kinases. (D) Cells were transfected with a control plasmid (control) or vectors expressing GFP-CLK1 (mCLK1), GFP-CLK2 (mCLK2), GFP-CLK3 (mCLK3), or GFP-CLK4 (mCLK4). Forty-eight hours post-transfection, cells were harvested and extracts fractionated on SDS-PAGE gels. Resultant blots were probed first with anti-GFP antibody to detect individual CLKs then with anti-tubulin antibody to confirm equal loading of samples.

**Figure 2:**
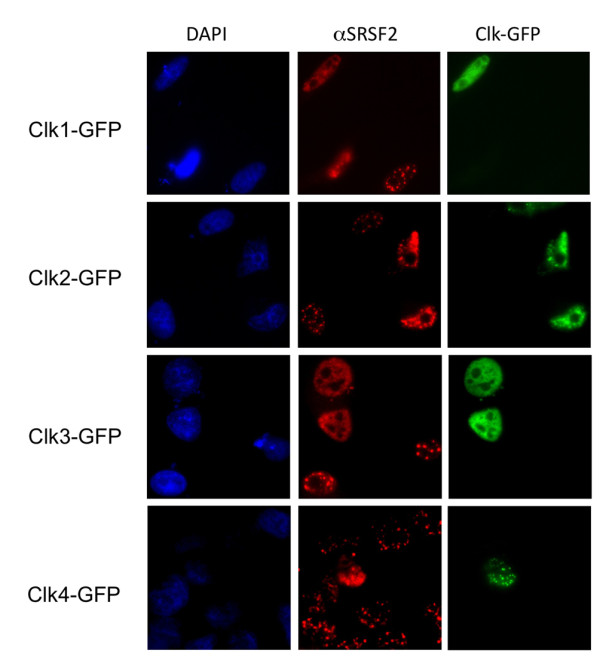
**Effect of CLK Overexpression on SRSF2 (SC35) Subcellular Distribution**. Cells were transfected with GFP-CLK expression vectors, incubated for forty-eight hours post-transfection, and then fixed and processed for immunofluorescence localization of SRSF2. Cells were stained with anti-SRSF2 antibody followed by Texas Red-conjugated donkey anti-mouse antibody and nuclei stained with DAPI. Shown are representative samples of the localization patterns of the CLKs and SRSF2 observed from > 5 experiments. Magnification 630x.

Multiple cellular kinases have been implicated in the phosphorylation of SR proteins, a modification critical to their function in RNA splicing [33,36-40]. To explore the potential roles of the members of the Cdc2-like kinase (CLK) family in the regulation of HIV-1 RNA processing and expression, GFP-tagged expression vectors for each of the CLKs were transfected into cells carrying an integrated, doxycycline-inducible form of HIV-1 (see additional file 1, Figure S1). As shown in Figure 1, the CLKs are a highly related family of proteins with CLK1 and CLK4 displaying the highest degree of similarity [26]. Subsequent analysis of cell lysates confirmed expression of each of the CLKs in this cell system (Figure 1D). Parallel examination of the effect of CLK overexpression on SRSF2 (SC35) subcellular distribution confirmed, as previously documented, that all of these factors disrupted SRSF2 subnuclear distribution from being primarily confined to nuclear speckles to being dispersed throughout the nucleus (Figure 2) as a result of the hyperphosphorylation of the protein [31,32,36].

**Figure 3:**
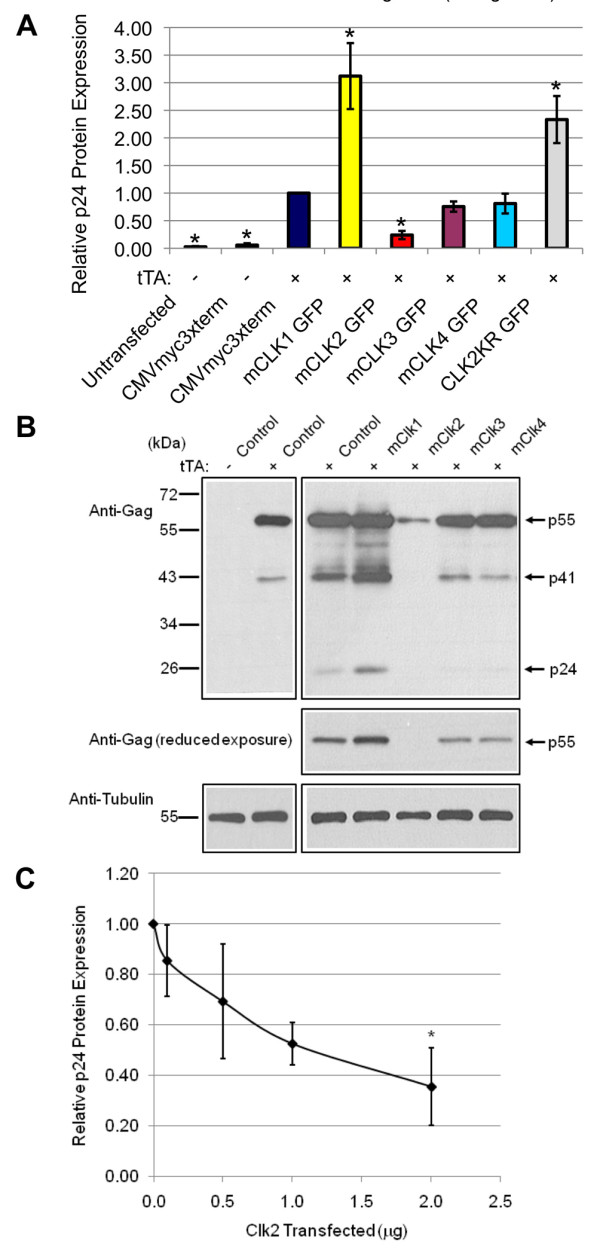
**Differential Effect of CLKs on HIV-1 Provirus Expression**. Cells were transfected with CMVmyc 3xTerm (-) or CMVtTa (+, to induce endogenous HIV-1 provirus expression) along with control plasmid (CMVmyc 3xterm) or vectors expressing GFP-CLK1, GFP-CLK2, GFP-CLK3, GFP-CLK4, or the kinase-inactive form, GFP-CLK2 KR. Forty-eight hours post-transfection, media and cells were harvested and Gag (p24) protein levels determined by (A) p24 ELISA or (B) western blot. Shown are the averaged results of >5 independent assays with asterisks denoting results determined to significantly different from control (+tTA) at a p value of < 0.05. (C) As above but cells were transfected with increasing amounts of CLK2 expression vector. Media was harvested two days post transfection and HIV-1 Gag (p24) protein levels determined by ELISA. Shown is the average of multiple independent trials (N = 3).

**Figure 4:**
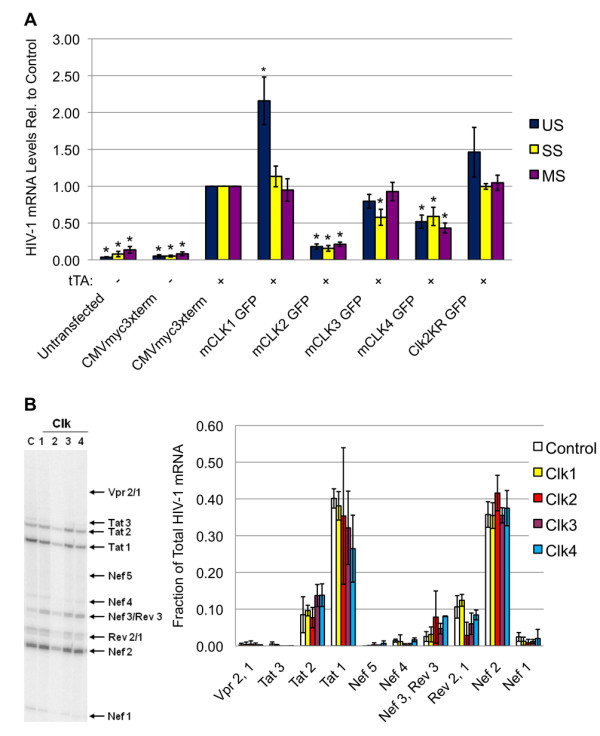
**Differential Effect of CLK Overexpression on HIV-1 RNA Accumulation and Splicing**. Cells were transfected with CMVmyc 3xTerm (-) or CMVtTa (+, to induce provirus expression) along with control plasmid (CMVmyc 3xterm) or vectors expressing GFP-CLK1, GFP-CLK2, GFP-CLK3 or GFP-CLK4. Forty-eight hours post-transfection, cells were harvested and total RNA extracted. (A) Abundance of unspliced (US), singly spliced (SS), and multiply spliced (MS) viral RNAs was determined by qRT-PCR as outlined in "Materials & Methods". Shown are the average of >7 independent analyses. (B) To examine the effect of overexpression of individual CLK proteins on viral RNA splicing, radioactive RT-PCR was performed on MS viral RNAs. Products were fractionated on 8M urea-PAGE gels and gels exposed to phosphor screens to detect the different splice products. For explanation of the products generated, please refer to additional file 2, Figure S2. On the left is a representative RT-PCR gel of the pattern observed and on the right, a summary of the relative abundance of each splice product (fraction of total viral MS RNA) for >3 independent assays. Asterisks denote values determined to be significantly different from control at a p value < 0.05.

The cell line, used in this study to examine the effect of CLK overexpression on virus replication, was generated by stable insertion of an HIV-1 provirus whose expression from the TetON promoter, within the viral LTR, is dependent on addition of doxycycline to activate the endogenous TetO transactivator (rtTA) or transfection with the constitutively active TetO transcriptional activator (tTA) [41,42] (see additional file 1, Figure S1). As shown in Figures 3A and 3B, cells transfected without the tTA activator did not express any HIV-1 Gag (p24) over background. Comparison of HIV-1 Gag expression of cells transfected with the various CLKs versus control vector revealed marked differences in response. Expression of GFP-CLK1 was observed to induce a ~3 fold increase in HIV-1 Gag expression while CLK 2 reduced synthesis of the same viral protein by ~4 fold. CLK3 and CLK4 overexpression had only modest effects. Expression of a catalytically inactive form of CLK2 (CLK2 KR) was observed to enhance Gag expression in this system consistent with it having a transdominant effect. Subsequent experiments determined that inhibition of HIV-1 was correlated with the level of CLK2 overexpression (Figure 3C). To explore the basis for the observed responses, total RNA was isolated from the cells and the abundance of HIV-1 unspliced (US), singly spliced (SS) and multiply spliced (MS) RNAs determined by qRT-PCR. As shown in Figure 4A, changes in viral Gag expression in response to the various CLKs correlated with changes in abundance of the respective mRNA: CLK1 overexpression increasing HIV-1 US RNA ~2 fold while CLK2 reduced accumulation of all HIV-1 RNAs by ~5 fold. CLK3 and CLK4 were observed to have more moderate effects on HIV-1 gene expression and viral RNA abundance. To assess whether CLK overexpression was also associated with any alterations in splice site selection, a radioactive RT-PCR was performed on the MS class of viral RNAs that detects the complete spectrum of MS RNA products generated (Figure 4B). These experiments revealed only subtle changes in relative abundance of MS RNA products in the presence of CLK2. Consequently, although CLK1 and CLK2 overexpression alters the extent of HIV-1 RNA accumulation, they do not cause a gross alteration in use of any specific set of splice sites.

### Chlorhexidine Inhibits CLK2, 3 and 4 Function and Alters HIV-1 RNA Processing

**Figure 5:**
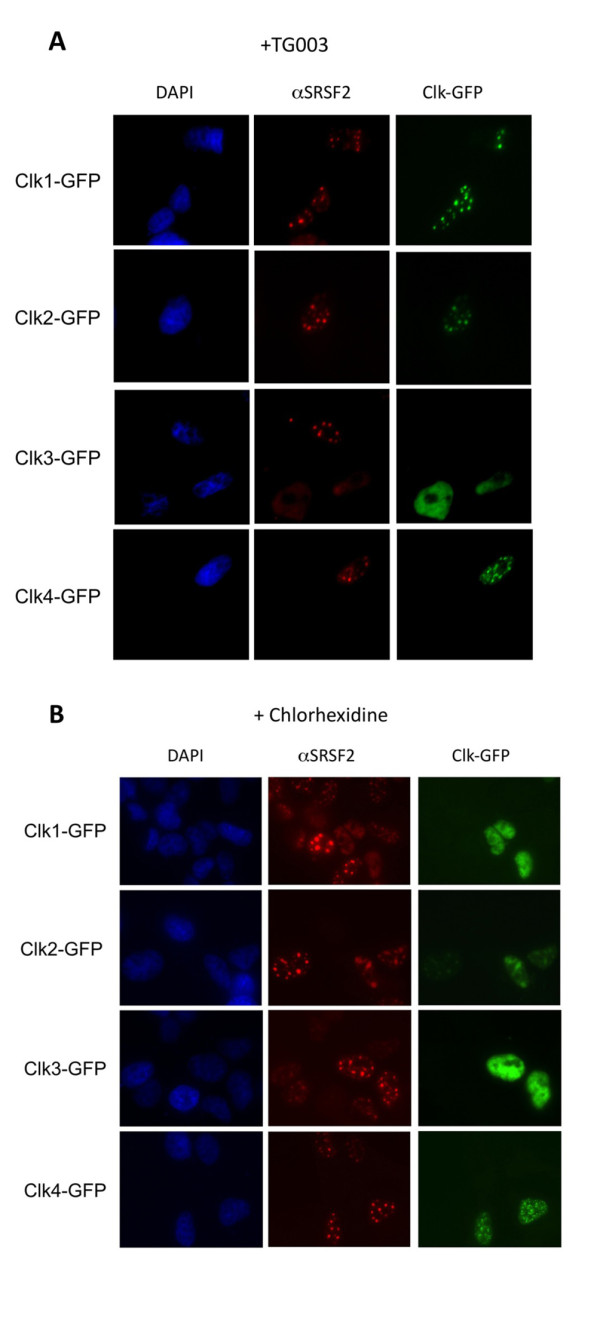
**TG003 and Chlorhexidine Alter the Effect of CLK Kinases on SRSF2 (SC35) Subcellular Distribution**. Cells were transfected with GFP-CLK expression vectors. Forty-eight hours post-transfection, cells were treated with (A) 10 μM TG003 or (B) 10 μM chlorhexidine for 4-5 h then fixed and processed for immunofluorescence localization of SRSF2. Cells were stained with anti-SRSF2 antibody followed by Texas Red conjugated donkey anti-mouse antibody and nuclei stained with DAPI. Shown are representative samples of the localization patterns of the CLKs and SRSF2 observed. Magnification 630x.

Our demonstration that altering the relative levels of CLKs has profound effects on HIV-1 gene expression suggested that we might be able to produce similar responses using CLK inhibitors. Recent work has identified two compounds that can alter the function of a specific subset of CLKs: TG003 inhibits CLK1, CLK4, and, to a lesser extent, CLK2, while chlorhexidine is an inhibitor of CLK2, 3,.and 4 in vitro [34,35]. To verify the activity of these compounds at doses used in subsequent assays, we examined their ability to suppress the disruption of nuclear speckles upon CLK overexpression by blocking hyperphosphorylation of SR proteins. As shown in Figure 5A, treatment of cells with TG003 was found to block disruption of nuclear speckles by CLK1, 2 and 4. In contrast, chlorhexidine prevented nuclear speckle disruption upon overexpression of CLK2, 3 and 4 (Figure 5B). In the case of both TG003 and chlorhexidine, drug treatment also resulted in movement of the affected CLKs (with the exception of CLK3) to subnuclear structures that partially overlap with nuclear speckles. An inactive analog of TG003 (TG009) was found to have no effect (data not shown), confirming that the response is attributable to specific effects of the inhibitors and not the solvent on CLK activity [34]. These observations confirm that TG003 and chlorhexidine have overlapping but different target protein specificities.

**Figure 6:**
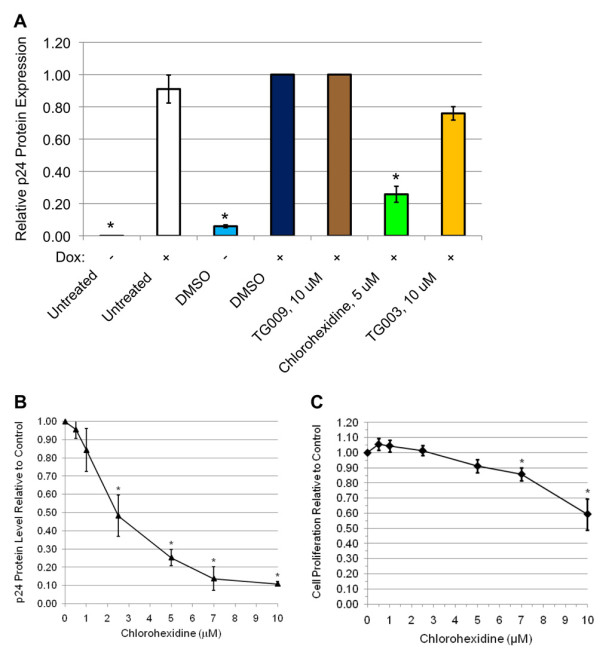
**Chlorhexidine is a Potent Inhibitor of HIV-1 Gene Expression**. (A) Cells were treated for 4-5 h with TG003, TG009 or chlorhexidine, then doxycycline was added to induce expression of the endogenous HIV-1 provirus. Twenty-four hours later, cell media were harvested, and HIV-1 Gag (p24) protein levels determined by ELISA. Shown are the average of >9 independent assays, asterisks denoting results determined to significantly different from control (DMSO +Dox.) at a p value < 0.01. (B) As described in A, but cells were treated with varying doses of chlorhexidine to identify the minimum dose required to suppress HIV-1 gene expression. (C) To assess the effect of chlorhexidine on cell viability, cells were incubated with indicated dose of chlorhexidine (0.5 - 10 μM) for 24 h then an XTT assay performed. Level of XTT conversion, which measures the number and metabolic activity of the cells, was compared to DMSO-treated cells.

**Figure 7:**
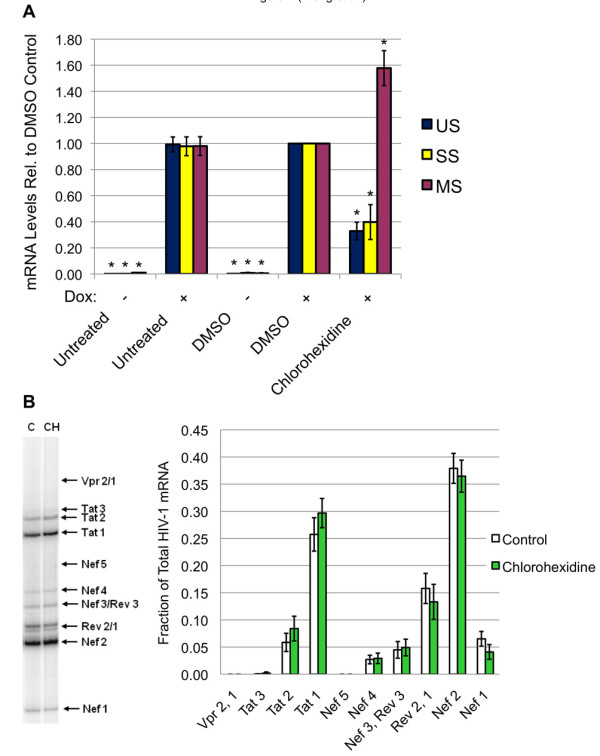
**Effect of Chlorhexidine on HIV-1 RNA Accumulation and Splicing**. Cells were treated for 4-5 h with TG003, TG009 or chlorhexidine (5 μM), then doxycycline was added to induce expression of the endogenous HIV-1 provirus. Twenty-four hours later, cells were harvested and total RNA extracted. (A) Abundance of US, SS, and MS viral RNAs was determined by qRT-PCR as outlined in "Materials & Methods". Shown are the average of >5 independent analyses. (B) To examine the effect of drug treatment on viral RNA splicing, radioactive RT-PCR was performed on MS viral RNAs and products fractionated on 8 M urea-PAGE gels followed by exposure to phosphor screens to detect the different splice products. On the left is a representative gel of the pattern observed and on the right, a summary of the relative abundance of each splice product over multiple assays (n > 6). Asterisks denote values determined to be significantly different from control at a p value < 0.05.

These compounds were subsequently evaluated for their effect on HIV-1 gene expression. HeLa cells containing the Tet-ON HIV-1 provirus were exposed to compounds for 4-5 hours prior to induction of the provirus by addition of doxycycline to the medium. Media, RNA and protein were harvested 24 h later. As shown in Figure 6A, neither TG003 nor TG009 had any substantial effect on induction of HIV-1 Gag protein, while chlorhexidine caused a ~4 fold reduction in expression of this viral protein. Evaluation of the dose-response characteristics of chlorhexidine on HIV-1 gene expression determined that significant repression occurred at doses of ~ 2.5 μM (Figure 6B). In contrast, examination of cell viability following chlorhexidine treatment (Figure 6C) revealed little effect at doses required to suppress virus replication in the time frame of the assay. Evaluation of the effect of chlorhexidine on HIV-1 RNA levels determined that it induced an alteration in viral RNA abundance, decreasing US and SS RNA accumulation by ~60% while increasing levels of MS RNAs 1.6 fold (Figure 7A). Parallel examination of viral MS RNA splicing patterns (Figure 7B, additional files 2 &3, Figure S2, 3) determined that neither chlorhexidine nor TG003 induced any significant alteration in use of specific splice sites within this MS class of HIV-1 RNAs.

### Chlorhexidine Treatment Inhibits HIV-1 Rev Accumulation

**Figure 8:**
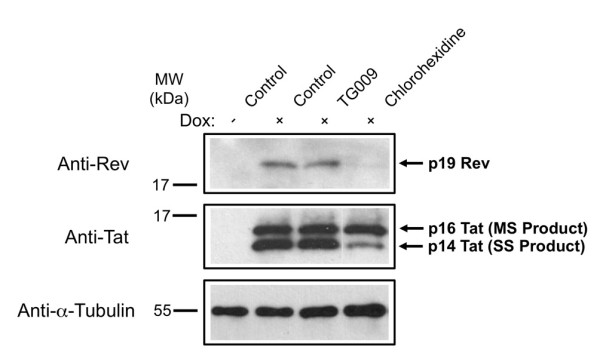
**Chlorhexidine Reduces Expression of HIV-1 Rev**. Cells were untreated or treated for 4-5 h with TG009 or chlorhexidine, then expression of the endogenous HIV-1 provirus was induce by addition of doxycycline. Twenty-four hours later, cells were harvested and cell extracts fractionated on SDS-PAGE gels. Resultant blots generated were probed with anti-Rev, anti-Tat or anti-tubulin antibodies to assess the effect of drugs on viral protein expression. Results shown are representative of >3 independent trials.

In addition to the alteration in HIV-1 RNA levels that could account for the loss of viral protein expression, we examined whether chlorhexidine treatment changed expression of any of the viral regulatory proteins such as Tat or Rev. Since Tat is essential for optimal HIV-1 promoter function and Rev is necessary for export of incompletely spliced viral RNAs to the cytoplasm [4-7], reduced expression of either or both would dramatically alter expression of the HIV-1 provirus. Western blots of extracts prepared from cells incubated in the presence or absence of drug (TG009, chlorhexidine) were probed to assess Tat and Rev expression. As shown in Figure 8, treatment of cells with chlorhexidine resulted in a marked reduction in Rev expression (to below the level of detection) without affecting levels of Tat p16 (encoded by MS RNA). However, Tat p14 levels were reduced upon chlorhexidine treatment. Since Rev is required for nuclear export of all HIV-1 US and SS RNAs, loss of Rev would be expected to reduce expression of all proteins encoded by this group of RNAs, including Tat p14 (encoded by SS RNA). Therefore, the observed changes in Tat expression can be directly related to the effect of chlorhexidine on Rev.

### Chlorhexidine is an Inhibitor of HIV-1 Replication in PBMCs

**Figure 9:**
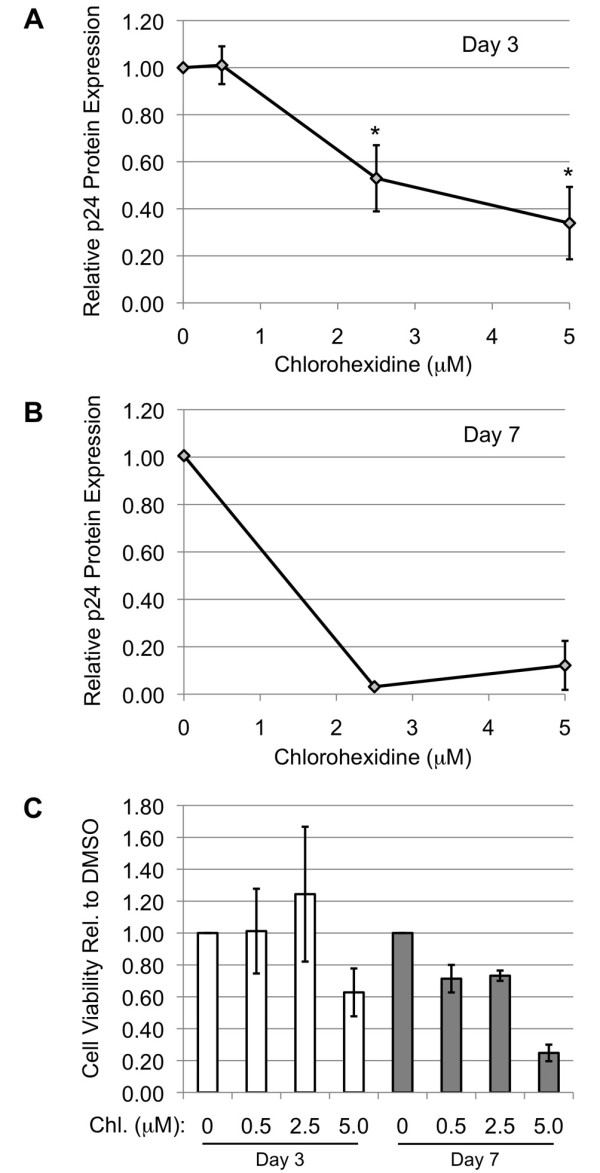
**Chlorhexidine Inhibits HIV-1 Replication in PBMCs**. To assess the effect of chlorhexidine on HIV-1 replication, PBMCs were infected with the BaL strain of HIV-1 for 2 h. Subsequently, varying doses of chlorhexidine (0.5-5 μM) were added to the medium. Medium -/+ drug was harvested (A) 3 and (B) 7 days post-infection and level of virus production determined by p24 ELISA. (C) The effect of chlorhexidine treatment on PBMC viability was monitored by trypan blue exclusion on days 3 and 7 post HIV-1 infection.

Our observation that chlorhexidine can dramatically reduce expression of the HIV-1 provirus in the TetON HIV cell line suggested the exciting possibility that it would be a potent inhibitor of HIV-1 replication in its natural context, CD4+ T cells. To test this hypothesis, PBMCs were infected with an R5 strain of HIV-1 (BaL) and cells subsequently treated with a range of chlorhexidine concentrations. Three and seven days post-infection, cell supernatants were harvested and levels of viral production determined by p24 (Gag) ELISA. As shown in Figure 9A and 9B, treatment of cells with doses of chlorhexidine 2.5 μM or greater resulted in a marked reduction in viral replication at both time points analyzed. Parallel measurement of cell viability over the same time period (Figure 9C) determined that doses of chlorhexidine required to suppress HIV-1 replication resulted in some reduction in cell viability over the course of this assay but not enough to account for the loss of virus replication.

## Discussion

Previous studies on the regulation of RNA processing have clearly outlined the important role of SR proteins in modulating usage of particular splicing sites [15]. Consequently, modulating RNA processing could be achieved by either regulating the abundance of specific SR proteins or modulating their activity through changes in the extent of their phosphorylation [43,44]. Either hyper- or hypo-phosphorylation of SR proteins has been shown to alter their ability to support RNA splicing in vitro and results in changes in alternative splicing choices [25-33,45]. Previous analysis of the role played by SRPK2 in controlling HIV-1 gene expression determined that its overexpression increased virus production putatively by modulating the activity of SRp75 [22]. Our findings on the effect of CLK overexpression on HIV-1 replication revealed that overexpression of individual CLKs had very distinct effects on HIV-1 RNA processing and gene expression. This point is best illustrated by comparison of CLK1 with CLK2. While CLK1 overexpression resulted in increased HIV-1 Gag RNA levels and protein synthesis, CLK2 overexpression led to dramatic suppression of HIV-1 Gag production associated with reduced accumulation of all viral RNAs (US, SS and MS). Differences between the various CLKs occurred despite comparable expression and their indistinguishable effects on SRSF2 subnuclear distribution (Figures 1 and 2). Previous analyses had demonstrated that altering CLK expression levels modified a number of alternative splicing events but, in most instances, changing expression of different CLKs had the same effect on the RNA splicing event being monitored [46-48]. Consequently, our observation of marked differences in effect of individual CLKs on HIV-1 is one of the first demonstrations of distinct activities between these individual family members. The contrasting effects of CLK2 versus the kinase inactive CLK2 KR (acting as a dominant negative) on HIV-1 expression confirm that the effects observed are due to kinase activity and not simply overexpression of the protein.

The basis for the differences between the various CLKs is presently unclear. Preliminary analysis of changes in SR protein phosphorylation upon overexpression of different CLKs revealed increased levels of SR protein phosphorylation but no differences in the SR proteins modified (data not shown). Consequently, differences may reflect differing extents of phosphorylation or modifications of specific residues within SR proteins. Alternatively, given that CLKs have been shown to interact with proteins outside of the SR protein family, the different activities could reflect phosphorylation of other host factors [36,49-52].

Similar to the differential responses to the overexpression of individual CLKs, the two CLK inhibitors tested also yielded very distinct effects. TG003 inhibits predominately CLK1 and CLK4, with reduced effect on CLK2 but had little to no effect on HIV-1 gene expression. In contrast, chlorhexidine, which blocked HIV-1 Gag and Rev synthesis, is a potent inhibitor of CLK3 and 4 with reduced activity against CLK2 as measured by its capacity to reverse CLK-induced effects on SRSF2 subnuclear distribution [35]. The fact that both drugs have overlapping activity against the various CLKs but yield strikingly different effects on virus expression suggests that the regulation of HIV-1 is due to the effect of chlorhexidine on CLK3 function alone, alterations in the relative activities of the individual CLKs present, or possibly modulation of another host factor. Altering relative activities among the individual CLKs by overexpression or drug inhibition could account for the inhibitory effects on HIV-1 gene expression.

Subsequent evaluation of the basis for chlorhexidine suppression of HIV-1 gene expression/replication indicated that the response is distinct from that seen upon overexpression of CLK2 or 3, as indicated by their effects on viral RNA accumulation. CLK3 overexpression had a limited effect on viral RNA abundance and CLK2 overexpression repressed accumulation of all HIV-1 RNAs. In contrast, chlorhexidine induced a shift in viral RNA accumulation, reducing levels of US and SS RNAs while increasing MS RNA levels. Such a response is suggestive of an enhancement of the overall splicing of HIV-1 RNAs given that subsequent analysis did not detect any shift in splice site usage. In addition, chlorhexidine selectively reduced Rev protein expression without any change in Tat (p16) levels. In the absence of any reduction in Rev RNA abundance, it would appear that chlorhexidine also selectively inhibits Rev RNA translation or reduces the stability of this protein. Whatever the mechanism, the reduced levels of Rev account for the loss of p24 (Gag) seen since Rev is essential to the export and translation of the corresponding viral US and SS RNAs.

## Conclusions

In summary, our experiments have highlighted the different activities among members of the CLK family in the regulation of HIV-1 gene expression and RNA processing. This finding suggests that these kinases likely selectively modulate alternative RNA splicing in the context of other genes. More significantly, we have demonstrated that small molecule inhibitors of specific CLKs can suppress HIV-1 gene expression and replication. Given that the process affected by chlorhexidine is distinct from those targeted by current HIV-1 therapeutics (i.e. entry, reverse transcription, integration, virus maturation), these findings highlight the feasibility of targeting viral RNA processing as a novel strategy to control HIV-1 replication that could be used in concert with current drug combinations to enhance the control of this infection [53]. Chlorhexidine is already in use in humans as the active ingredient in mouthwash and topical antiseptics at doses (~2 mM) 1000 fold above those required to block HIV-1 replication. While the observed toxicity of chlorhexidine in the context of PBMCs precludes its systemic use, its application on mucosal surfaces is well tolerated in humans suggesting the use of chlorhexidine as a microbicide to block HIV-1 transmission at the site of entry (mucosal surfaces) by inhibiting virus replication in the local region or reducing the level of virus secretion at these surfaces in those already infected. A better understanding of the mechanism of chlorhexidine/CLK2 action will likely provide greater insights that could guide the development of additional compounds with improved specificity and activity.

## Materials and methods

### Plasmids and cell tranfections

To explore the effects of CLKs on HIV-1 protein expression/RNA, HeLa cells stably transduced with an inducible Tet-On HIV-1 system were used [41,42]. Activation of HIV-1 gene expression was achieved by either addition of doxycyline (Dox) at a concentration of 2 μg/ml or transfection with the constitutively active Tet activator, tTA. Modification of the published HIV Tet-ON system consisted of deleting the RT and IN genes by *Mls1 *digestion and using the resulting construct to generate the HeLa rtTA HIVΔmls cell line by retroviral transduction and cloning (Figure S1). To explore the effect of CLK overexpression on HIV-1 gene expression, cells were transfected with empty expression plasmid (CMVmyc 3xterm) or vectors expressing GFP-CLK1, GFP-CLK2, GFP-CLK3, GFP-CLK4 (provided by J. Bell, University of Ottawa) or GFP-CLK2 KR (provided by S. Stamm, University of Kentucky) along with CMVtTA to induce provirus expression in cells taking up DNA. Transfections were performed using polyethylene imine (PEI, Polysciences Inc.). Cells and media were harvested 48 h post-transfection to assess effects on HIV-1 gene expression.

In the case of drug treatment, cells were seeded onto 6-well plates at approximately 0.5 × 10^6 ^cells per well (~50-75% confluence) in IMDM with 10% FBS and antibiotics (1 × Pen-Strep, 100 μg/mL, 1 × Amphotericin B, 0.5 μg/mL) (Wisent Corporation). Drugs were obtained from Sigma-Aldrich (Chlorhexidine, cat. #C6143)) or provided by Masatoshi Hagiwara (TG003/TG009, Tokyo Medical & Dental University) and solubilized to 10 mM with DMSO. After 4-5 hours of drug treatment, HIV expression was induced by addition of doxycycline (2 μg/ml final concentration). After approximately 24 hours, cell supernatants were harvested for p24 ELISA, while cells were harvested for RNA or protein analyses. Cell viability was monitored by either trypan blue exclusion (Gibco) or XTT assay (Sigma-Aldrich) [54].

### Analysis of HIV-1 protein expression

For analysis of HIV-1 (Gag) protein expression, media was adjusted to 1% Triton X-100 and assayed by p24 ELISA as described in the HIV-1 p24^CA ^antigen capture assay kit (AIDS & Cancer virus Program, NCI-Frederick, Frederick, MD, USA).

### Quantitation of HIV-1 mRNA Levels

Cells were harvested by incubation in 2 mM EDTA-PBS for 15 minutes at 37°C and pelleted cells used in RNA purification or protein analysis. RNA was purified by Aurum Total RNA Mini Kits (Bio-Rad, Cat. #732-6820). Purified RNA was reverse transcribed using M-MLV (Invitrogen, Cat. #28025-013). cDNAs reactions (20 μl) were diluted to 150 μL and used in qRT-PCR analysis of HIV-1 mRNA levels using the standard curve method. Each reaction was set-up as follows: 0.4 μL of Taq DNA polymerase (5 U/μL, NEB, Cat. #M0267L), 2.5 μL of ThermolPol buffer, 2.5 μL of 10X SYBR Green I (Sigma-Aldrich, Cat. #S9430), 2.5 μL of 2.5 mM dNTPs, 1.0 μL of 5' primer (0.1 ug/uL), and 1.0 μL of 3' primer (0.1 μg/μL), 10.1 μL H_2_O, and 5 μL of cDNA. The forward and reverse primers used in the quantitation of HIV-1 mRNA are shown below: unspliced (US), 5' - GAC GCT CTC GCA CCC ATC TC - 3' and 5' - CTG AAG CGC GCA CGG CAA - 3'; singly spliced (SS), 5' - GGC GGC GAC TGG AAG AAG C - 3' and 5' - CTA TGA TTA CTA TGG ACC ACA C - 3'; and multiply spliced (MS), 5' - GAC TCA TCA AGT TTC TCT ATC AAA - 3' and 5' - AGT CTC TCA AGC GGT GGT - 3'. Results were normalized to the housekeeping gene, ß-actin, which served as an internal loading control. 5'-GAGCGGTTCCGCT GCCCTGAGGCACTC-3' and 5'-GGGCAGTGATCTCCTTCTGCATCCTG-3'. qRT-PCRs were run on an Eppendorf Mastercycler ep realplex^4^. The cycle conditions used for US, MS, and Actin were 95°C, 2 min followed by 40 cycles of 95°C, 15s; 60°C, 15s; and 72°C, 15s. SS conditions were 95°C, 2 min followed by 40 cycles of 95°C, 30s; 55°C, 30s; and 72°C, 30s.

### Analysis of HIV-1 alternative splicing

The effect of CLKs and drugs on HIV-1 splice site usage was performed as previously described [55]. cDNAs were analyzed for changes in splice site usage within the 2 kb, MS RNA class. The forward and reverse primers are as followed: 5'-GGGCAGTGATCTCCTTCTGCATCCTG -3' and 5' -TCA TTG CCA CTG TCT TCT GCT CT - 3'. Initial rounds of cold RT-PCR were set-up as followed: 1 μL cDNA, 1 μL of Taq DNA polymerase, 5 μL of 10X ThermolPol buffer, 4 μL of 2.5 mM dNTPs, 10 μL of forward primer (10 μM), 10 μL of reverse primer (10 μM), and 19 μL of H_2_O in a 50 μL final reaction volume. Thermocycler conditions used were 95°C, 2 min followed by 34 cycles of 95°C, 1 min; 57°C, 1 min; and 68°C, 1 min; and ended with 68°C, 5 min; and 4°C, indefinitely. A second round of radioactive PCR was run with the following changes/additions to the conditions described above: 3 μL of diluted cDNA from the first PCR reaction (1/10^th ^dilution), 0.5 μL of α-^32^P-dCTP (Perkin Elmer, #BLU013A250UC), and 16.5 μL of H_2_O. The same thermocycler conditions were also used except only 5 cycles were run. An equal volume of loading buffer (90% formamide, 10 mM EDTA, 0.025% xylene cyanol, and 0.025% bromophenol blue) was added to the products and heated at 95°C for 5 minutes prior to loading onto sequencing gels (6% polyacrylamide, 8 M Urea, 1xTBE), and products detected by phosphoimager. Densitometry was performed using ImageJ by density plots generated for each lane. Values for each HIV-1 RNA specie(s) detected were divided by the total density from all viral RNA species in a sample (fraction of total RNA).

### Western blot analysis of HIV-1 proteins

Cells pellets were solubilized in 100 to 300 uL of RIPA buffer (50 mM Tris-HCl pH 7.5, 150 mM NaCl, 1% NP-40, 0.5% sodium deoxycholate, 0.1% SDS), incubated at 95°C for 5 minutes, and centrifuged for 2 minutes at 12 K × g. Lysate supernatants were quantitated by Bradford assay. For Western blotting, equal amounts of protein were run on 7, 10 or 12% SDS-PAGE, transferred to PVDF (0.45 μm, Perkin-Elmer, Cat. #NEF1002) by electrophoretic transfer and blots blocked in 5% Milk-PBS-T (0.05% Tween-20, 1xPBS) for 1 h at room temperature. For Rev, blots were probed with a 1/250 dilution of mouse monoclonal (Rev-6) antibody to HIV-1 Rev (Abcam, Cat. #ab85529) in PBS-T. For α-Tubulin, blots were washed then probed with mouse monoclonal α-Tubulin antibody (Sigma-Aldrich, Cat. #T9026) diluted to 1/5000 in PBS-T. For Tat, blots were probed with a 1/5000 dilution of rabbit polyclonal antibody to HIV-1 Tat (Abcam, Cat. #ab43014) in 5% Milk-PBS-T. After primary antibody incubations, blots were washed in PBS-T and then incubated with a 1/5000 dilution of isotype-specific HRP-conjugated secondary antibody in 5% Milk-PBS-T available from Jackson ImmunoResearch (Cat. #715-036-150 for rabbit and Cat. #711-036-152 for mouse). After washes, blots were developed using Western Lightning ECL (Perkin-Elmer, Cat. #NEL101) and exposed to autoradiography film.

### Effect of drugs on CLK kinase modulation of SR protein subnuclear distribution

Cells were transfected with vectors expressing GFP-tagged CLK1, CLK2, CLK3 or CLK4. Two days post-transfection, cells were either treated with DMSO, TG003 or chlorhexidine for 4-5 h or overnight prior to fixation in 4% paraformaldehyde, 1xPBS. Cells were subsequently permeabilized by treatment with 1% Triton X-100, 1xPBS followed by blocking in 3% BSA, 1xPBS for 1 h. Subcellular distribution of SRSF2 (SC35) was determined by staining with a mouse anti-SRSF2 (SC35) antibody (BD Sciences) followed by incubation with a Texas Red-labeled donkey anti-mouse antibody (Jackson Immunoresearch). Cells were stained with DAPI prior to mounting to detect nuclei. Images were captured using a Leica DMR microscope.

### HIV-1 infection of PBMCs

Blood was isolated from HIV seronegative donors, leukapheresed, and stored at -80°C. PBMCs were isolated from blood of healthy donors using Ficoll-Hypaque (VWR, Cat. #CA95038-170L as detailed by manufacturer. For infections, 1 mL of cells (100 × 10^6^) were thawed, diluted in R-2 (RPMI containing 2% FBS) and centrifuged at 300 RCF for 10 minutes at room temperature. Thawed cells were cultured at 37°C in R-10 or RPMI complete medium (10% FBS (heat-inactivated), 1% GlutaMAX-1 (Invitrogen, Gibco, Cat. #35050-061), 1x Pen-Strep (100 μg/mL; Wisent Corp.), and 1 × Amphotericin B (0.5 μg/mL; Wisent Corp.), containing 2 μg/mL of PHA-L (Sigma, Cat. #L2769) and 20 U/mL of IL-2 (BD Pharmingen, Cat. #554603). After 48 h, PBMCs were isolated by Ficoll-Hypaque density gradient centrifugation, washed with R-2 medium, and centrifuged at 450 RCF for 25 min. to remove dead cells. Next, cells from each donor were resuspended in R-10 with 20 U/mL of IL-2 and infected with a R5 HIV-1 strain (BaL) at an MOI of 10^-2^. After 2 h of infection at 37°C, cells were washed 3 times with R-2. Cell pellets were resuspended in R-10 with 20 U/mL of IL-2, seeded at 2 × 10^6 ^cells per well in 24-well plates, and treated with drugs at the indicated doses. Media were harvested every 2 or 3 days after infection and assayed by p24 ELISA as described. The effect of these drugs on cell viability was assessed by trypan blue stain and counted on Kova Glasstic slide 10 with grids (VWR, Cat. #CA36200-020).

## Competing interests

The authors wish to indicate that they have no competing interests.

## Authors' contributions

RW was responsible for performing all experiments and analyses herein which include examining the effect of CLK overexpression and drug treatments on HIV-1 gene expression, replication, RNA accumulation, and splice site selection. AB contributed data on the effect of chlorhexidine on HIV-1 RNA accumulation/cell viability and AM provided data on the effect of overexpression of CLK2 KR on viral gene expression. Both WD and SGO provided HIV-1 BaL virus and training in studies examining the effect of chlorhexidine on HIV-1 replication in PBMCs. AC and RW were involved in the design and coordination of the experiments as well as preparing the manuscript for submission. All authors read and approved the final manuscript.

## Supplementary Material

Additional file 1 **Figure S1. Characterization of HeLa HIVrtTA ΔMLS Cell Line**. (A) Outline of the HIV-1 proviral construct used to generate cell line. Provirus has insertion of TetO operator sites in the U3 region, substitution of Nef with the doxycyclline-dependent transactivator rtTA, mutational inactivaion of Tat and TAR and deletion of the RT and IN reading frames. HeLa cells were transduced and screened for doxycycline dependent expression of the HIV-1 structural proteins. (B) Cells were incubated in the presence or absence of doxycycline (Dox), fixed with paraformaldehyde then Gag protein expression detected using anti-Gag antibodies. (C-E) Following incubation for 24 h in the presence or absence of doxycycline, cells were harvested for RNA (C) or protein (D, E) extracted and fractionated on gels. (C) Following transfer to nitrocellulose, northern blots were probed with radioalabelled probe to the HIV-1 LTR (allowing detection of HIV-1 US, SS and MS RNAs) or endogenous GAPDH RNA. (D,E) Proteins were fractionated on SDS-PAGE gels, blotted, and blots probed with antibodies against HIV-1 Gag (p55. p41, p24), Env (gp160, gp120), Tat (p16, p14), or Rev (p19). To confirm equivalent loading, blots were also probed with antibody to α-tubulin (Tub). In (D), cells were treated with doxycycline or transfected with plasmid encoding the doxycycline-independent transactivator, tTA. Click here for file

Additional file 2 **Figure S2. Outline of HIV-1 RNA Alternative Splicing **Shown at the top is the organization of the HIV-1 proviral genome, indicating the position of the multiple 5' splice sites (SD1 to SD4) and 3' splice sites (SA1 to SA7) used. Below is an illustration of the spliced RNAs generated by processing of the HIV-1 genomic RNA. Indicated are the common (open boxes) and alternative (closed boxes) exons used in the generation of the SS (4 kb) and MS (1.8 kb) viral RNAs. At the bottom, is the nomenclature used in reference to the exon composition of the individual RNAs generated for both the SS and MS classes of HIV-1 RNAs. Click here for file

Additional file 3 **Figure S3**. **Effect of TG003 and TG009 on HIV-1 RNA Splicing **To examine the effect of drug treatment on viral RNA splicing, radioactive RT-PCR was performed for MS viral RNAs and products fractionated on 8 M urea-PAGE gels followed by exposure to phosphor screens to detect the different splice products. Shown is a summary of the relative abundance of each splice product over multiple assays relative to untreated (control) cells. Click here for file

## References

[B1] Stoltzfus CM Madsen JM Role of viral splicing elements and cellular RNA binding proteins in regulation of HIV-1 alternative RNA splicing Current HIV Research 2006 4 1 43 55 10.2174/157016206775197655 16454710

[B2] Tazi J Bakkour N Marchand V Ayadi L Aboufirassi A Branlant C Alternative splicing: regulation of HIV-1 multiplication as a target for therapeutic action Febs J 2010 277 4 867 876 10.1111/j.1742-4658.2009.07522.x 20082634

[B3] McLaren M Marsh K Cochrane A Modulating HIV-1 RNA processing and utilization Front Biosci 2008 13 5693 5707 18508616 10.2741/3110

[B4] Nekhai S Jeang KT Transcriptional and post-transcriptional regulation of HIV-1 gene expression: role of cellular factors for Tat and Rev Future Microbiol 2006 1 417 426 10.2217/17460913.1.4.417 17661632

[B5] Romani B Engelbrecht S Glashoff RH Functions of Tat: the versatile protein of human immunodeficiency virus type 1 J Gen Virol 2010 91 Pt 1 1 12 19812265 10.1099/vir.0.016303-0

[B6] Pollard V Malim M The HIV-1 Rev Protein Ann. Rev. Microbiol 1998 52 491 532 10.1146/annurev.micro.52.1.491 9891806

[B7] Hope TJ The ins and outs of HIV Rev Arch. Biochem. Biophys 1999 365 186 191 10.1006/abbi.1999.1207 10328811

[B8] Madsen JM Stoltzfus CM A suboptimal 5' splice site downstream of HIV-1 splice site A1 is required for unspliced viral mRNA accumulation and efficient virus replication Retrovirology 2006 3 10 10.1186/1742-4690-3-10 16457729 PMC1403798

[B9] Kammler S Otte M Hauber I Kjems J Hauber J Schaal H The strength of the HIV-1 3' splice sites affects Rev function Retrovirology 2006 3 89 10.1186/1742-4690-3-89 17144911 PMC1697824

[B10] Bilodeau PS Domsic JK Mayeda A Krainer AR Stoltzfus CM RNA splicing at human immunodeficiency virus type 1 3' splice site A2 is regulated by binding of hnRNP A/B proteins to an exonic splicing silencer element J. Virol 2001 75 18 8487 8497 10.1128/JVI.75.18.8487-8497.2001 11507194 PMC115094

[B11] Amendt B Si Z Stoltzfus CM Presence of Exon Splicing Silencers within Human Immunodeficiency Virus Type 1 tat Exon 2 and tat-rev Exon 3: Evidence for Inhibition Mediated by Cellular Factors Mol. Cell. Biol 1995 15 4606 4615 7623852 10.1128/mcb.15.8.4606 PMC230701

[B12] Damgaard CK Tange TO Kjems J hnRNP A1 controls HIV-1 mRNA splicing through cooperative binding to intron and exon splicing silencers in the context of a conserved secondary structure RNA 2002 8 1401 1415 10.1017/S1355838202023075 12458794 PMC1370347

[B13] Marchand V Mereau A Jacquenet S Thomas D Mougin A Gattoni R Stevenin J Branlant C A Janus splicing regulatory element modulates HIV-1 tat and rev mRNA production by coordination of hnRNP A1 cooperative binding J. Mol. Biol 2002 323 4 629 652 10.1016/S0022-2836(02)00967-1 12419255

[B14] Zhu J Mayeda A Krainer A Exon identity established through differential antagonism between exonic splicing silencer-bound hnRNP A1 and enhancer-bound SR proteins Mol. Cell 2001 8 6 1351 1361 10.1016/S1097-2765(01)00409-9 11779509

[B15] Graveley BR Sorting out the complexity of SR protein functions RNA 2000 6 9 1197 1211 10.1017/S1355838200000960 10999598 PMC1369994

[B16] Hallay H Locker N Ayadi L Ropers D Guittet E Branlant C Biochemical and NMR study on the competition between proteins SC35, SRp40, and heterogeneous nuclear ribonucleoprotein A1 at the HIV-1 Tat exon 2 splicing site J Biol Chem 2006 281 48 37159 37174 10.1074/jbc.M603864200 16990281

[B17] Madsen JM Stoltzfus CM An exonic splicing silencer downstream of the 3' splice site A2 is required for efficient human immunodeficiency virus type 1 replication J Virol 2005 79 16 10478 10486 10.1128/JVI.79.16.10478-10486.2005 16051840 PMC1182660

[B18] Caputi M Freund M Kammler S Asang C Schaal H A bidirectional SF2/ASF- and SRp40-dependent splicing enhancer regulates human immunodeficiency virus type 1 rev, env, vpu, and nef gene expression J. Virol 2004 78 12 6517 6526 10.1128/JVI.78.12.6517-6526.2004 15163745 PMC416506

[B19] Jablonski JA Caputi M Role of cellular RNA processing factors in human immunodeficiency virus type 1 mRNA metabolism, replication, and infectivity J Virol 2009 83 2 981 992 10.1128/JVI.01801-08 19004959 PMC2612387

[B20] Ropers D Ayadi L Gattoni R Jacquenet S Damier L Branlant C Stevenin J Differential effects of the SR proteins 9G8, SC35, ASF/SF2 and SRp40 on the utilization of the A1 to A5 splicing sites of HIV-1 RNA J Biol Chem 2004 279 29963 29973 10.1074/jbc.M404452200 15123677

[B21] Jacquenet S Decimo D Muriaux D Darlix JL Dual effect of the SR proteins ASF/SF2, SC35 and 9G8 on HIV-1 RNA splicing and virion production Retrovirology 2005 2 1 33 10.1186/1742-4690-2-33 15907217 PMC1180853

[B22] Ryo A Suzuki Y Arai M Kondoh N Wakatsuki T Hada A Shuda M Tanaka K Sato C Yamamoto M Identification and characterization of differentially expressed mRNAs in HIV type 1-infected human T cells AIDS Res Hum Retroviruses 2000 16 10 995 1005 10.1089/08892220050058416 10890361

[B23] Maldarelli F Xiang C Chamoun G Zeichner SL The expression of the essential nuclear splicing factor SC35 is altered by human immunodeficiency virus infection Virus Res 1998 53 1 39 51 10.1016/S0168-1702(97)00130-5 9617768

[B24] Fukuhara T Hosoya T Shimizu S Sumi K Oshiro T Yoshinaka Y Suzuki M Yamamoto N Herzenberg LA Hagiwara M Utilization of host SR protein kinases and RNA-splicing machinery during viral replication Proc Natl Acad Sci USA 2006 103 30 11329 11333 10.1073/pnas.0604616103 16840555 PMC1544086

[B25] Stojdl DF Bell JC SR protein kinases: the splice of life Biochem Cell Biol 1999 77 4 293 298 10.1139/o99-046 10546892

[B26] Bullock AN Das S Debreczeni JE Rellos P Fedorov O Niesen FH Guo K Papagrigoriou E Amos AL Cho S Kinase domain insertions define distinct roles of CLK kinases in SR protein phosphorylation Structure 2009 17 3 352 362 10.1016/j.str.2008.12.023 19278650 PMC2667211

[B27] Ngo JC Chakrabarti S Ding JH Velazquez-Dones A Nolen B Aubol BE Adams JA Fu XD Ghosh G Interplay between SRPK and Clk/Sty kinases in phosphorylation of the splicing factor ASF/SF2 is regulated by a docking motif in ASF/SF2 Mol Cell 2005 20 1 77 89 10.1016/j.molcel.2005.08.025 16209947

[B28] Colwill K Feng LL Yeakley JM Gish GD Caceres JF Pawson T Fu XD SRPK1 and Clk/Sty protein kinases show distinct substrate specificities for serine/arginine-rich splicing factors J Biol Chem 1996 271 40 24569 24575 10.1074/jbc.271.40.24569 8798720

[B29] Velazquez-Dones A Hagopian JC Ma CT Zhong XY Zhou H Ghosh G Fu XD Adams JA Mass spectrometric and kinetic analysis of ASF/SF2 phosphorylation by SRPK1 and Clk/Sty J Biol Chem 2005 280 50 41761 41768 10.1074/jbc.M504156200 16223727

[B30] Nayler O Schnorrer F Stamm S Ullrich A The cellular localization of the murine serine/arginine-rich protein kinase CLK2 is regulated by serine 141 autophosphorylation J Biol Chem 1998 273 51 34341 34348 10.1074/jbc.273.51.34341 9852100

[B31] Duncan PI Stojdl DF Marius RM Bell JC In vivo regulation of alternative pre-mRNA splicing by the Clk1 protein kinase Mol Cell Biol 1997 17 10 5996 6001 9315658 10.1128/mcb.17.10.5996 PMC232448

[B32] Duncan PI Stojdl DF Marius RM Scheit KH Bell JC The Clk2 and Clk3 dual-specificity protein kinases regulate the intranuclear distribution of SR proteins and influence pre-mRNA splicing Exp Cell Res 1998 241 2 300 308 10.1006/excr.1998.4083 9637771

[B33] Koizumi J Okamoto Y Onogi H Mayeda A Krainer AR Hagiwara M The subcellular localization of SF2/ASF is regulated by direct interaction with SR protein kinases (SRPKs) J Biol Chem 1999 274 16 11125 11131 10.1074/jbc.274.16.11125 10196197

[B34] Muraki M Ohkawara B Hosoya T Onogi H Koizumi J Koizumi T Sumi K Yomoda J Murray MV Kimura H Manipulation of alternative splicing by a newly developed inhibitor of Clks J Biol Chem 2004 279 23 24246 24254 10.1074/jbc.M314298200 15010457

[B35] Younis I Berg M Kaida D Dittmar K Wang C Dreyfuss G Rapid-response splicing reporter screens identify differential regulators of constitutive and alternative splicing Mol Cell Biol 2010 30 7 1718 1728 10.1128/MCB.01301-09 20123975 PMC2838070

[B36] Colwill K Pawson T Andrews B Prasad J Manley JL Bell JC Duncan PI The Clk/Sty protein kinase phosphorylates SR splicing factors and regulates their intranuclear distribution Embo J 1996 15 2 265 275 8617202 PMC449941

[B37] Gui JF Tronchere H Chandler SD Fu XD Purification and characterization of a kinase specific for the serine- and arginine-rich pre-mRNA splicing factors Proc Natl Acad Sci USA 1994 91 23 10824 10828 10.1073/pnas.91.23.10824 7526381 PMC45118

[B38] Nayler O Stamm S Ullrich A Characterization and comparison of four serine- and arginine-rich (SR) protein kinases Biochem J 1997 326 Pt 3 693 700 9307018 10.1042/bj3260693 PMC1218723

[B39] Nikolakaki E Simos G Georgatos SD Giannakouros T A nuclear envelope-associated kinase phosphorylates arginine-serine motifs and modulates interactions between the lamin B receptor and other nuclear proteins J Biol Chem 1996 271 14 8365 8372 10.1074/jbc.271.14.8365 8626534

[B40] Rossi F Labourier E Forne T Divita G Derancourt J Riou JF Antoine E Cathala G Brunel C Tazi J Specific phosphorylation of SR proteins by mammalian DNA topoisomerase I Nature 1996 381 6577 80 82 10.1038/381080a0 8609994

[B41] Zhou X Vink M Berkhout B Das AT Modification of the Tet-On regulatory system prevents the conditional-live HIV-1 variant from losing doxycycline-control Retrovirology 2006 3 82 10.1186/1742-4690-3-82 17094796 PMC1637113

[B42] Zhou X Vink M Klaver B Verhoef K Marzio G Das AT Berkhout B The genetic stability of a conditional live HIV-1 variant can be improved by mutations in the Tet-On regulatory system that restrain evolution J Biol Chem 2006 281 25 17084 17091 10.1074/jbc.M513400200 16627480

[B43] Yeakley JM Tronchere H Olesen J Dyck JA Wang HY Fu XD Phosphorylation regulates in vivo interaction and molecular targeting of serine/arginine-rich pre-mRNA splicing factors J Cell Biol 1999 145 3 447 455 10.1083/jcb.145.3.447 10225947 PMC2185075

[B44] Xiao SH Manley JL Phosphorylation of the ASF/SF2 RS domain affects both protein-protein and protein-RNA interactions and is necessary for splicing Genes Dev 1997 11 3 334 344 10.1101/gad.11.3.334 9030686

[B45] Prasad J Colwill K Pawson T Manley JL The protein kinase Clk/Sty directly modulates SR protein activity: both hyper- and hypophosphorylation inhibit splicing Mol Cell Biol 1999 19 10 6991 7000 10490636 10.1128/mcb.19.10.6991 PMC84694

[B46] Eisenreich A Bogdanov VY Zakrzewicz A Pries A Antoniak S Poller W Schultheiss HP Rauch U Cdc2-like kinases and DNA topoisomerase I regulate alternative splicing of tissue factor in human endothelial cells Circ Res 2009 104 5 589 599 10.1161/CIRCRESAHA.108.183905 19168442

[B47] Hartmann AM Rujescu D Giannakouros T Nikolakaki E Goedert M Mandelkow EM Gao QS Andreadis A Stamm S Regulation of alternative splicing of human tau exon 10 by phosphorylation of splicing factors Mol Cell Neurosci 2001 18 1 80 90 10.1006/mcne.2001.1000 11461155

[B48] Yomoda J Muraki M Kataoka N Hosoya T Suzuki M Hagiwara M Kimura H Combination of Clk family kinase and SRp75 modulates alternative splicing of Adenovirus E1A Genes Cells 2008 13 3 233 244 10.1111/j.1365-2443.2008.01163.x 18298798

[B49] Rodgers JT Haas W Gygi SP Puigserver P Cdc2-like kinase 2 is an insulin-regulated suppressor of hepatic gluconeogenesis Cell Metab 2010 11 1 23 34 10.1016/j.cmet.2009.11.006 20074525 PMC2807620

[B50] Stoilov P Daoud R Nayler O Stamm S Human tra2-beta1 autoregulates its protein concentration by influencing alternative splicing of its pre-mRNA Human Molecular Genetics 2004 13 5 509 524 10.1093/hmg/ddh051 14709600

[B51] Kojima T Zama T Wada K Onogi H Hagiwara M Cloning of human PRP4 reveals interaction with Clk1 J Biol Chem 2001 276 34 32247 32256 10.1074/jbc.M103790200 11418604

[B52] Moeslein FM Myers MP Landreth GE The CLK family kinases, CLK1 and CLK2, phosphorylate and activate the tyrosine phosphatase, PTP-1B J Biol Chem 1999 274 38 26697 26704 10.1074/jbc.274.38.26697 10480872

[B53] Flexner C HIV drug development: the next 25 years Nat Rev Drug Discov 2007 6 12 959 966 10.1038/nrd2336 17932493

[B54] Jost LM Kirkwood JM Whiteside TL Improved short- and long-term XTT-based colorimetric cellular cytotoxicity assay for melanoma and other tumor cells J Immunol Methods 1992 147 2 153 165 1548398 10.1016/s0022-1759(12)80003-2

[B55] Purcell D Martin MA Alternative splicing of human immunodeficiency virus type 1 mRNA modulates viral protein expression, replication, and infectivity J Virol 1993 67 6365 6378 8411338 10.1128/jvi.67.11.6365-6378.1993 PMC238071

