# Supplementary figures and images for: Differential effect of CLK SR Kinases on HIV-1 gene expression: potential novel targets for therapy
Source: Retrovirology. 2011 Jun 17;8:47. doi: 10.1186/1742-4690-8-47 (PMC3148977; doi:10.1186/1742-4690-8-47)

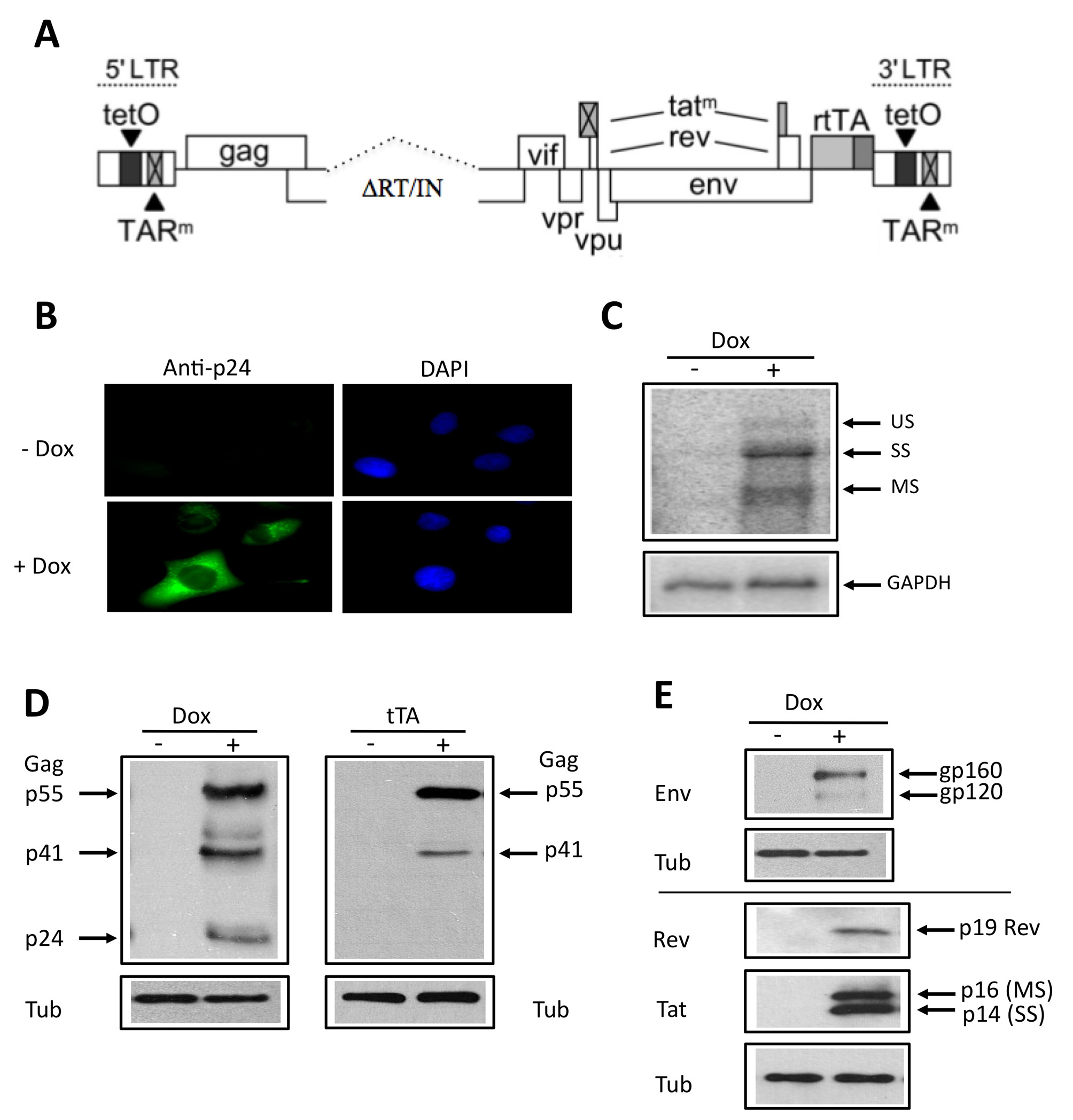

Supplement: Additional file 1 — Figure S1. Characterization of HeLa HIVrtTA ΔMLS Cell Line. (A) Outline of the HIV-1 proviral construct used to generate cell line. Provirus has insertion of TetO operator sites in the U3 region, substitution of Nef with the doxycyclline-dependent transactivator rtTA, mutational inactivaion of Tat and TAR and deletion of the RT and IN reading frames. HeLa cells were transduced and screened for doxycycline dependent expression of the HIV-1 structural proteins. (B) Cells were incubated in the presence or absence of doxycycline (Dox), fixed with paraformaldehyde then Gag protein expression detected using anti-Gag antibodies. (C-E) Following incubation for 24 h in the presence or absence of doxycycline, cells were harvested for RNA (C) or protein (D, E) extracted and fractionated on gels. (C) Following transfer to nitrocellulose, northern blots were probed with radioalabelled probe to the HIV-1 LTR (allowing detection of HIV-1 US, SS and MS RNAs) or endogenous GAPDH RNA. (D,E) Proteins were fractionated on SDS-PAGE gels, blotted, and blots probed with antibodies against HIV-1 Gag (p55. p41, p24), Env (gp160, gp120), Tat (p16, p14), or Rev (p19). To confirm equivalent loading, blots were also probed with antibody to α-tubulin (Tub). In (D), cells were treated with doxycycline or transfected with plasmid encoding the doxycycline-independent transactivator, tTA. [file 1742-4690-8-47-S1.JPEG]

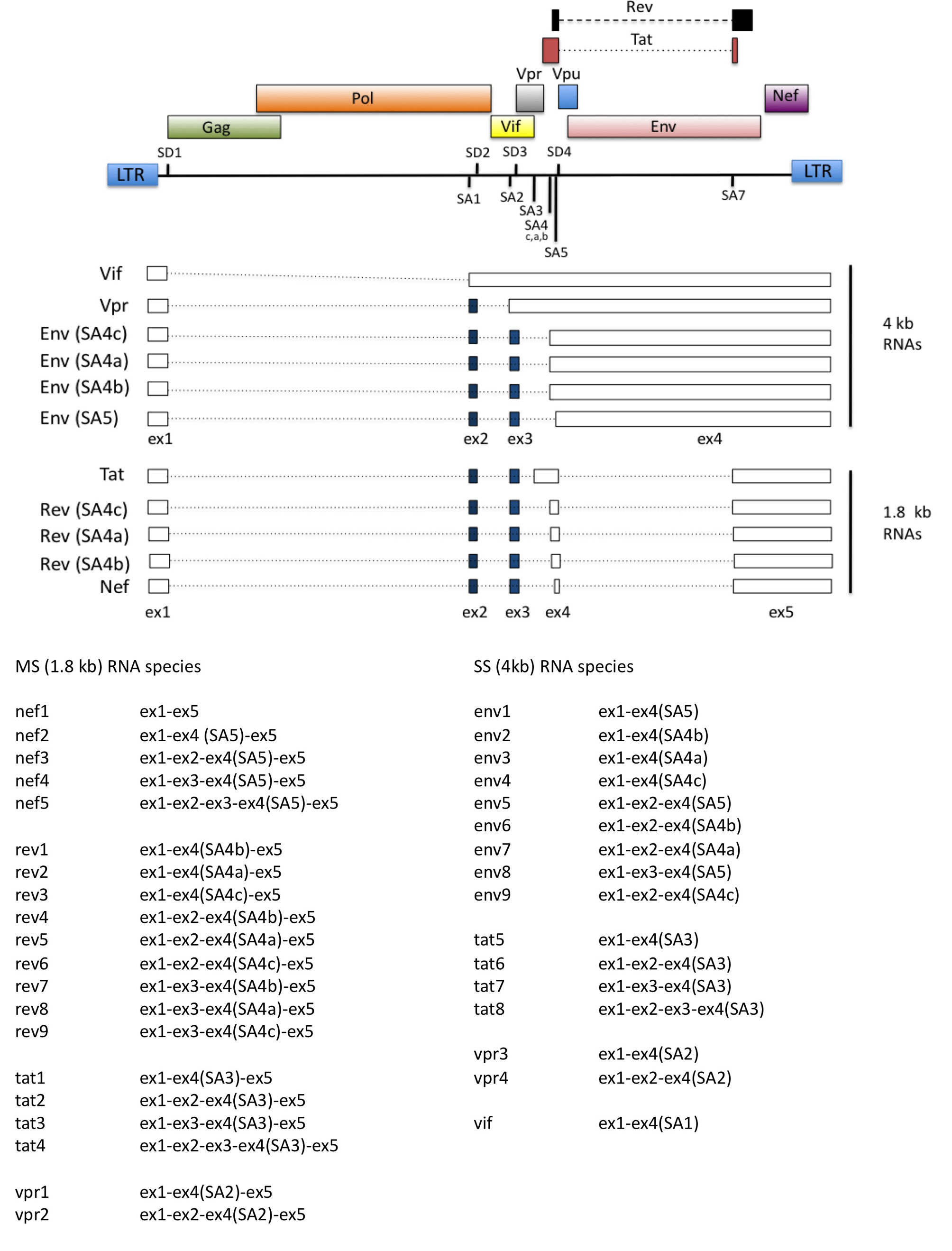

Supplement: Additional file 2 — Figure S2. Outline of HIV-1 RNA Alternative Splicing Shown at the top is the organization of the HIV-1 proviral genome, indicating the position of the multiple 5' splice sites (SD1 to SD4) and 3' splice sites (SA1 to SA7) used. Below is an illustration of the spliced RNAs generated by processing of the HIV-1 genomic RNA. Indicated are the common (open boxes) and alternative (closed boxes) exons used in the generation of the SS (4 kb) and MS (1.8 kb) viral RNAs. At the bottom, is the nomenclature used in reference to the exon composition of the individual RNAs generated for both the SS and MS classes of HIV-1 RNAs. [file 1742-4690-8-47-S2.JPEG]

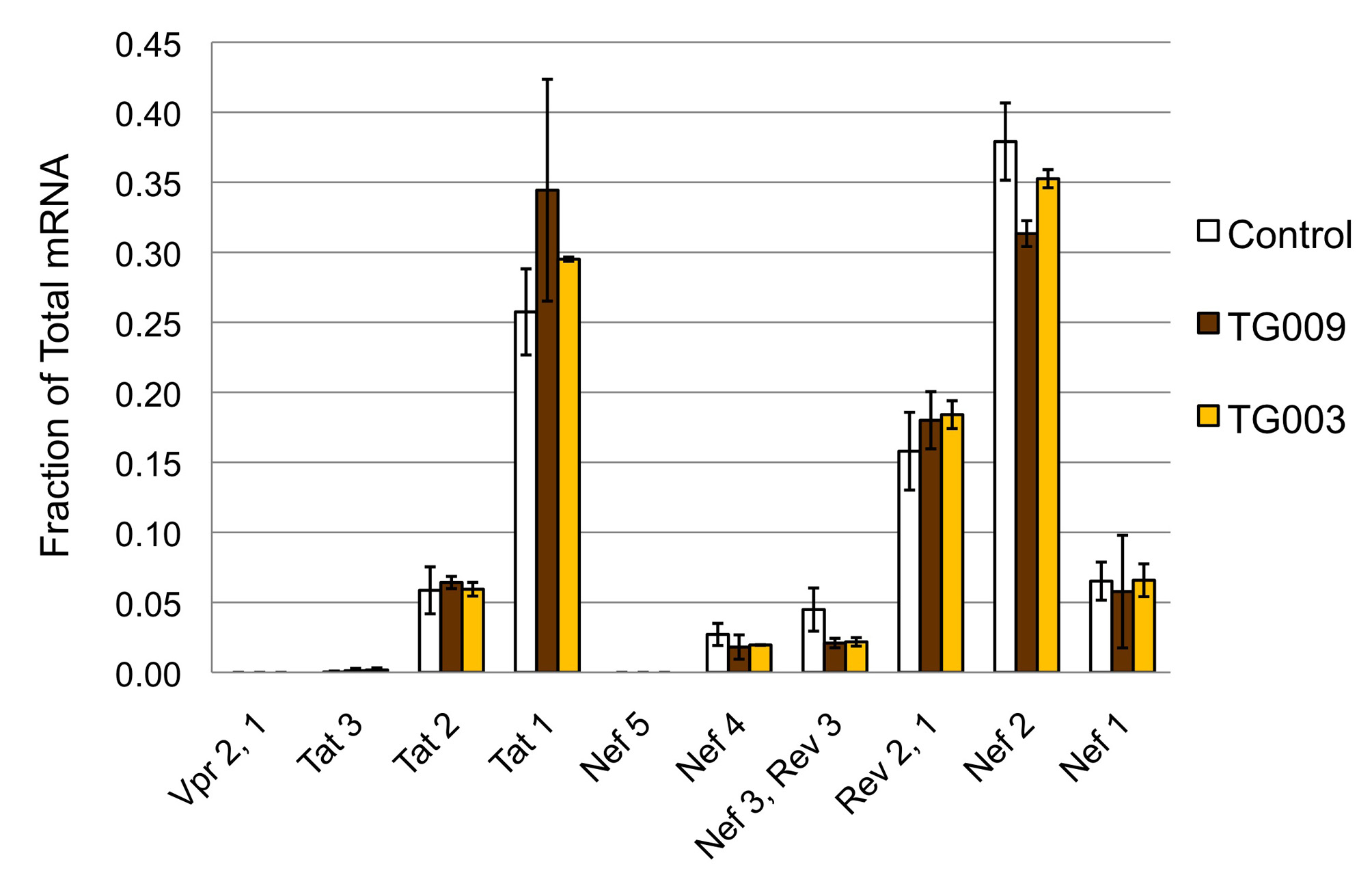

Supplement: Additional file 3 — Figure S3. Effect of TG003 and TG009 on HIV-1 RNA Splicing To examine the effect of drug treatment on viral RNA splicing, radioactive RT-PCR was performed for MS viral RNAs and products fractionated on 8 M urea-PAGE gels followed by exposure to phosphor screens to detect the different splice products. Shown is a summary of the relative abundance of each splice product over multiple assays relative to untreated (control) cells. [file 1742-4690-8-47-S3.JPEG]
